# Vitamin E (α-Tocopherol) Does Not Ameliorate the Toxic Effect of Bisphenol S on the Metabolic Analytes and Pancreas Histoarchitecture of Diabetic Rats

**DOI:** 10.3390/toxics11070626

**Published:** 2023-07-19

**Authors:** Sheila I. Peña-Corona, Dinorah Vargas-Estrada, Juan I. Chávez-Corona, C. Adriana Mendoza-Rodríguez, Sara Caballero-Chacón, José Pedraza-Chaverri, María Isabel Gracia-Mora, Diana Patricia Galván-Vela, Helena García-Rodríguez, Francisco Sánchez-Bartez, Marcela Vergara-Onofre, Gerardo Leyva-Gómez

**Affiliations:** 1 Departamento de Farmacia, Facultad de Química, Universidad Nacional Autónoma de México, Ciudad de México 04510, Mexico; 2 Departamento de Fisiología y Farmacología, Facultad de Medicina Veterinaria y Zootecnia, Universidad Nacional Autónoma de México, Ciudad de México 04510, Mexico; dinorahvestrada@fmvz.unam.mx (D.V.-E.); juan.isaac.chavez@gmail.com (J.I.C.-C.); saracachas@hotmail.com (S.C.-C.); 3 Departamento de Biología, Facultad de Química, Universidad Nacional Autónoma de México, Ciudad de México 04510, Mexico; adrimed@yahoo.com (C.A.M.-R.); pedraza@unam.mx (J.P.-C.); 4 Departamento de Química Inorgánica y Nuclear, Facultad de Química, Universidad Nacional Autónoma de México, Ciudad de México 04510, Mexico; isabel.gracia@quimica.unam.mx (M.I.G.-M.); franciscosbartez@quimica.unam.mx (F.S.-B.); 5 Unidad de Investigación Preclínica (UNIPREC), Facultad de Química, Universidad Nacional Autónoma de México, Ciudad de México 04510, Mexico; paty.gv@hotmail.com (D.P.G.-V.); helena82gr@gmail.com (H.G.-R.); 6 Departamento de Producción Agricola y Animal, Universidad Autónoma Metropolitana Unidad Xochimilco, Ciudad de México 04960, Mexico; mvergara@correo.xoc.uam.mx

**Keywords:** bisphenol S, endocrine-disrupting compounds, diabetic rats, vitamin E, biochemistry

## Abstract

This study investigated whether the coadministration of vitamin E (VitE) diminishes the harmful effects provoked by plasticizer bisphenol S (BPS) in the serum metabolites related to hepatic and renal metabolism, as well as the endocrine pancreatic function in diabetic male Wistar rats. Rats were divided into five groups (*n* = 5–6); the first group was healthy rats (Ctrl group). The other four groups were diabetic rats induced with 45 mg/kg bw of streptozotocin: Ctrl-D (diabetic control); VitE-D (100 mg/kg bw/d of VitE); BPS-D (100 mg/kg bw/d of BPS); The animals from the VitE + BPS-D group were administered 100 mg/kg bw/d of VitE + 100 mg/kg bw/d of BPS. All compounds were administered orally for 30 days. Body weight, biochemical assays, urinalysis, glucose tolerance test, pancreas histopathology, proximate chemical analysis in feces, and the activity of antioxidants in rat serum were assessed. The coadministration of VitE + BPS produced weight losses, increases in 14 serum analytes, and degeneration in the pancreas. Therefore, the VitE + BPS coadministration did not have a protective effect versus the harmful impact of BPS or the diabetic metabolic state; on the contrary, it partially aggravated the damage produced by the BPS. VitE is likely to have an additive effect on the toxicity of BPS.

## 1. Introduction

Bisphenol A (BPA) is an established endocrine disrupting (ED) compound used in the manufacture of polycarbonate plastics and epoxide resins [1]. The adverse effects of BPA are most often induced by oxidative stress and the dynamic balance of enzymatic antioxidants, in addition to the classical genomic and non-genomic mechanisms [2,3,4]. In recent years, BPA has been subject to more stringent regulations from international government organizations. As a result, the industry has increased the use of alternatives, such as bisphenol S (BPS), the primary alternative to BPA [5,6].

The annual BPS manufacture or import rate was 1000 to 10,000 tons in the ECHA report in 2015 [7]. BPS was detected as the second most frequent compound after BPA in food samples from the United States [8]. Additionally, BPS has been detected in Chinese river water and sediments [9,10]. In 2015, the presence of BPS was determined in surface water samples from China, India, Japan, and Korea [11]. Since BPS is found in the environment and is produced in quantities of thousands of tons, BPS can be considered omnipresent in the global environment.

Recent studies have shown that BPS alters serum metabolites, organ weights, and reproductive endpoints; moreover, it acts as an obesogen and has a potency of hormonal activities in the same order of magnitude and of similar action as BPA [12,13]. It has been reported that the toxicity of bisphenols may be exacerbated by a poor diet, metabolic disorders such as diabetes mellitus (DM), and coexisting diseases [14]. In addition, BPS modulates type 1 DM (T1DM) development [15]. However, much remains unknown about BPS’ potential toxicities, including its effect on T1DM.

According to the International Diabetes Federation Atlas, about 415 million people were estimated to have DM globally in 2015. That total has been projected to increase to 642 million by 2040 [16]. T1DM is an autoimmune disease characterized by pancreatic β-cell destruction and has been growing in incidence globally [17]. Genetic, nutritional, and environmental factors are known to be associated with the development of T1DM, including exposure to environmental obesogens [18].

Since the harmful effects of bisphenols as ubiquitous compounds are of great importance for human health, efforts have been made to propose alternatives to counteract or reduce the consequences of their exposure, such as the use of chitosan [19], micronutrients [20,21], and plants or extracts of these with antioxidant properties [22,23,24], as well as vitamins E (VitE), C and B [25,26,27].

Vitamins, like VitE, protect the cell membrane from oxidation and have potent cholesterol-lowering and antioxidant properties [28,29]. Although results about the effects of VitE in DM models are not conclusive, it has been reported that oral administration of VitE for three weeks reduced blood glucose levels in experimental T1DM Wistar rats [30]. Moreover, supplementation with antioxidants, such as VitE, may also benefit diabetic patients [31].

The protective effect of VitE on the toxicity produced by administering BPA has presented favorable results. In Wistar rats, 200 mg/kg body weight (bw) of VitE protected the muscle tissue and blood cells from changes in biochemical parameters and antioxidant imbalance produced by 20 mg/kg bw of BPA [32]. Male rats treated with 1000 mg/kg of VitE for five weeks also showed similar results [33]. Thus, their use as a compound that attenuates the harmful effects of BPS could be promising. Consequently, the study of VitE as a protector from the damaging effects produced by BPS in a DM model is of interest.

Worldwide, the number of people that suffer from DM is rapidly increasing. Since ubiquitous exposure to bisphenols can exacerbate the pathogenesis of the disease, this article aimed to investigate whether exposure to VitE can reduce the harmful effects produced by BPS in diabetic rats on the structure and function of the pancreas, activity of antioxidant enzymes, absorption of nutrients, and liver and kidney function using biochemical assessment, the area under the curve (AUC) as a glucose tolerance index, and proximate chemical analysis (PCA) in stools. To our knowledge, this is the first study to evaluate the effects of coadministration of VitE with BPS in a male rat diabetic model.

Contrary to what we expected, the results obtained in this trial suggest that coadministration of 100 mg/kg bw/d of VitE with 100 mg/kg bw/d of BPS for 30 days aggravates the damage induced by BPS to the diabetic metabolic state. 

## 2. Materials and Methods

### 2.1. Chemicals

Bisphenol S (BPS; Sigma-Aldrich Inc., Toluca, Mexico, CAS No. 80-09-1; purity of 99%); BPS was dissolved in olive oil Merainsa^®^ without antioxidants (vehicle) purchased from local commercial sources. VitE (α-tocopherol; Sigma-Aldrich Inc., Mexico, CAS No. 10191-41-0; purity of 100%). Streptozotocin (STZ; Sigma-Aldrich Inc., Mexico, CAS No. 18883-66-4; purity of ≥95% by HPLC). The name and references (brand, catalog number, lot, expiration) are shown in Table 1.

### 2.2. Experimental Design

**Figure 1:**
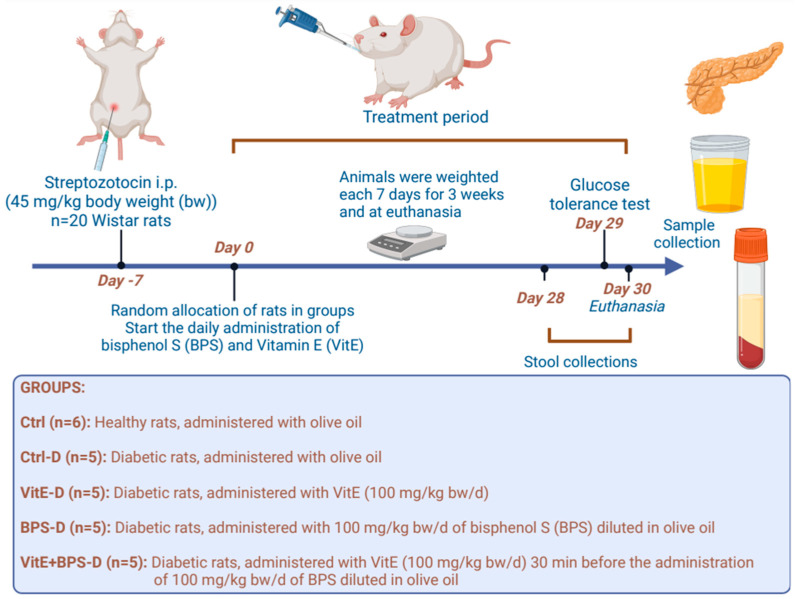
Experimental design.

A completely blind, randomized experiment with repeated measures over time was performed. The male rats were divided into five groups (*n* = 5–6) as follows (Figure 1).

Twenty-six male Wistar rats weighing 250–300 g from the Animal Facility of the Cell Physiology Institute of the National Autonomous University of Mexico (UNAM) in Mexico City were used. The Institutional Committee for the Care and Use of Laboratory Animals, Faculty of Chemistry, UNAM, Mexico, approved the experimental procedures for the Care and Use of Experimental Animals in the present article (Trade number: FQ/CICUAL/467/22). Also, all experimental methods were designed according to Mexican legislation NOM-062-ZOO-1999. All animals were housed in polycarbonate cages with stainless steel covers in a controlled temperature room at 20 °C (12 h light/dark cycle and relative humidity of 50 ± 10%). The animals remained for 14 days in acclimatization before the beginning of the experiment. Throughout the experiment, rats had free access to water and pellet laboratory chow BIO-DIETA-LAB 7300 (ABENE^®^, Atizapán de Zaragoza, Estado de México, Mexico). Guaranteed analysis: Raw protein, 23.5% min; Crude fat, 6% min; Raw fiber, 4% max; Ash, 8% max; Humidity, 12% max; Nitrogen free extract (NFE), 46.5%.

Diabetes was induced with an intraperitoneal injection of 45 mg/kg bw of streptozotocin (STZ). To confirm the diagnosis of diabetes, rats were checked 1 and 2 days after drug administration by measuring blood glucose with a commercial glucometer (OneTouch^®^ ultra mini-Johnson & Johnson, Milpitas, CA, USA) and test strips (OneTouch^®^ mini) after 4 h of fasting. Rats with values above 200 mg/dL glucose were considered diabetic. We started BPS and/or VitE administration 7 days after the streptozotocin (STZ) injection to ensure all rats treated with STZ were diabetic. All animals were weighed weekly in the first 3 weeks and on day 30. Doses were administered orally daily for 30 days between 9:00 and 11:00 a.m. In the VitE-BPS-D group, VitE was administered 30 min before BPS administration.

The dose of 100 mg/kg bw/d of BPS used in this study was chosen based on results from our group, in which we observed that this dose produced harmful effects in reproductive variables in Wistar rats in a chronic administration for 15 weeks (work in progress). Additionally, BPS administered to adult male Sprague Dawley rats increased serum glucose, total cholesterol, and triglycerides with 30, 60, and 120 mg/kg bw/daily for 30 days [34]. Also, 100 mg/kg bw/d of VitE protected against alterations produced by bisphenols in antioxidant enzymes and liver damage [35,36]. Moreover, it improved blood urea and creatinine levels and increased antioxidant enzyme activities in the kidney [37].

Stools for the proximate chemical analysis were obtained from massage in the perianal zone of animals each day for 3 days before euthanasia. In addition, we made a pool for each group to evaluate absorbed nutrients and digestibility.

The animals were weighed, and euthanasia was carried out under anesthesia with Ketamine (PISA^®^, Mexico City, México) 40–80 mg/kg plus Xylazine (PISA^®^, México) 5–10 mg/kg according to Al-Mousawi et al. (2010) [38], followed by decapitation on the day after the end of the treatment. The blood was immediately collected, and serum samples were obtained by centrifugation in a centrifuge: Bejman J221, rotor BejmanJA-18.1, for 15 min at 169× *g* at 4 °C and stored at −80 °C until use for antioxidant and biochemical analysis. At euthanasia, the pancreas was collected and fixed in 4% *w*/*v* paraformaldehyde in phosphate-buffered saline. Urine was obtained directly from the bladder with a sterile needle syringe, deposited in collecting tubes, and stored at −80° until use. The samples were randomly numbered by people who recorded data before technics, and investigators analyzed them. Until the statistical analysis, the results from the samples were grouped.

### 2.3. Antioxidant Enzyme Activity

The activities of glutathione peroxidase (GPx), glutathione reductase (GR), and glutathione-S-transferase (GST) in rat serum were measured according to Pérez-Rojas et al. 2011 [39]. The GPx activity was assessed by the disappearance of NADPH at 340 nm in a coupled assay containing H_2_O_2_, glutathione (GSH), and GR. GR activity was evaluated by disappearing NADPH at 340 nm in a reaction mixture containing oxidized glutathione as a substrate. GST activity was assayed at 340 nm in a mix determining GSH /1-chloro-2,4-dinitrobenzene (CDNB) complexes [39].

### 2.4. Glucose Tolerance Test

For the oral glucose tolerance test (OGTT), animals were fasted for 4 h (morning fast, from 8:00 a.m. until 12:00 p.m.), and blood samples were obtained from the tail vein. Animals were administered 2 g/kg bw of glucose by oral gavage, and blood samples were taken at 0, 15, 30, 45, 60, 90, and 120 min. The blood glucose was measured using blood glucose test strips (OneTouch^®^ mini) and a glucometer (OneTouch^®^ ultra mini-Johnson & Johnson). The AUC values were considered as glucose tolerance indexes.

### 2.5. Biochemical Assays

Albumin, alanine aminotransferase (ALT), albumin/globulin (A/G) ratio, aspartate aminotransferase (AST), total bilirubin, blood urea nitrogen (BUN), creatinine, globulin, high-density lipoprotein cholesterol (HDL-c), low-density lipoprotein cholesterol (LDL-c), total cholesterol, total protein, and urea were determined by enzymatic colorimetric assays in a DIRUI model CS-T240 auto-chemistry analyzer according to the manufacturer’s instructions. The parameters A/G ratio, globulin, and urea were calculated. These parameters are significantly affected in diabetic conditions [40] and BPS administration. Thus, we considered evaluating serum parameters to determine if the coadministration of VitE with BPS attenuated the damage produced by BPS. Additionally, we compared the results with clinical laboratory parameters for Wistar rats and other studies made with diabetic rats [41,42,43].

### 2.6. Urinalysis

Leukocytes, nitrites, urobilinogen, protein, pH, blood, specific gravity, ketones, bilirubin, glucose, and reactive strips were used for urine analysis (GIMA URS-10T (24076)). We read the reagent areas visually at the time specified in the color chart for a semi-quantitative result according to the manufacturer’s protocol [44]. We conducted a physical evaluation based on Queremel et al. (2022) [45].

### 2.7. Nutrient Absorption and Digestibility

Proximal feces and feed analyses were performed to evaluate the percentage of nutrients retained or absorbed in the animals’ digestive tracts. The PAC was made in the Animal Nutrition and Biochemistry Department, Faculty of Veterinary Medicine (UNAM) [46]. The insoluble ash in hydrochloric acid 2N (AIA) was obtained in feed pellets and stools [47]. To evaluate the digestibility, we used the formula: (100 − (% AIA in dry matter in feed/% AIA in dry weight in stools) × 100) + 20.20

### 2.8. Pancreas Histology

After fixation, the samples were dehydrated by increasing ethanol concentrations, cleared with xylene, and incorporated into paraffin. Pancreas tissue was cut to a thickness of 5 μm and mounted on glass slides. The cuts were then dewaxed into xylene, rehydrated by decreasing ethanol concentrations, and stained with hematoxylin and eosin dyes for histological examination. Twenty-six histological sections stained with the hematoxylin and eosin technique were reviewed in a LEICA MOPA-UNIPREC-01 bi-head optical microscope. All the tissue in the slide was evaluated by sliding the slide in a zigzag pattern, observing first with the 4× objective, followed by 10× and 40×. In some specific cases, 100× magnification was used. We evaluated damage grades with symbols: “−” and “+”. The symbol “−” indicated no damage; “+” indicated scarce damage; “++” was considered moderate damage; and “+++” was considered severe damage. To evaluate the pancreas morphometry, we took five photos of each animal’s sample in 4× objective and measured the area and number of Langerhans islets with the ImageJ program. Evaluation of the histological alterations was based on Zachary (2016) and Shubin et al. (2016) [48,49].

### 2.9. Statistical Analysis

All data were evaluated for normality with D’Agostino & Pearson test or the Shapiro–Wilk test when the number of samples was small. When data were normal, we used ANOVA and a correction for multiple comparisons by controlling the false discovery rate (FDR) using the two-stage step-up method of Benjamini, Krieger, and Yekutieli (q ≤ 0.05). When data were not normal, we used a generalized linear model (GLM) and a Tukey Kramer post hoc test. Body weight values and blood glucose were evaluated in two-way ANOVA, considering one factor the group and the other the treatment day. Additionally, the mean of all body weights obtained in each group during the treatment period was considered the total weight. We use GLM to compare the total percentage of loss or weight gain between groups. The Prism 2.01 program (Graph Pad, San Diego, CA, USA) was used to calculate ANOVA, and IBM^®^ SPSS Statistics version 28 was used to evaluate the non-normal data. A probability value of *p* ≤ 0.05 was considered significant. In the case of the FDR test, we considered it significant when the individual *p* value was ≤ 0.05 and when the program showed the comparisons as “discovery” (q ≤ 0.05). Data are expressed as mean ± standard error of the mean (SEM) unless otherwise stated.

## 3. Results

### 3.1. Body Weight

**Figure 2:**
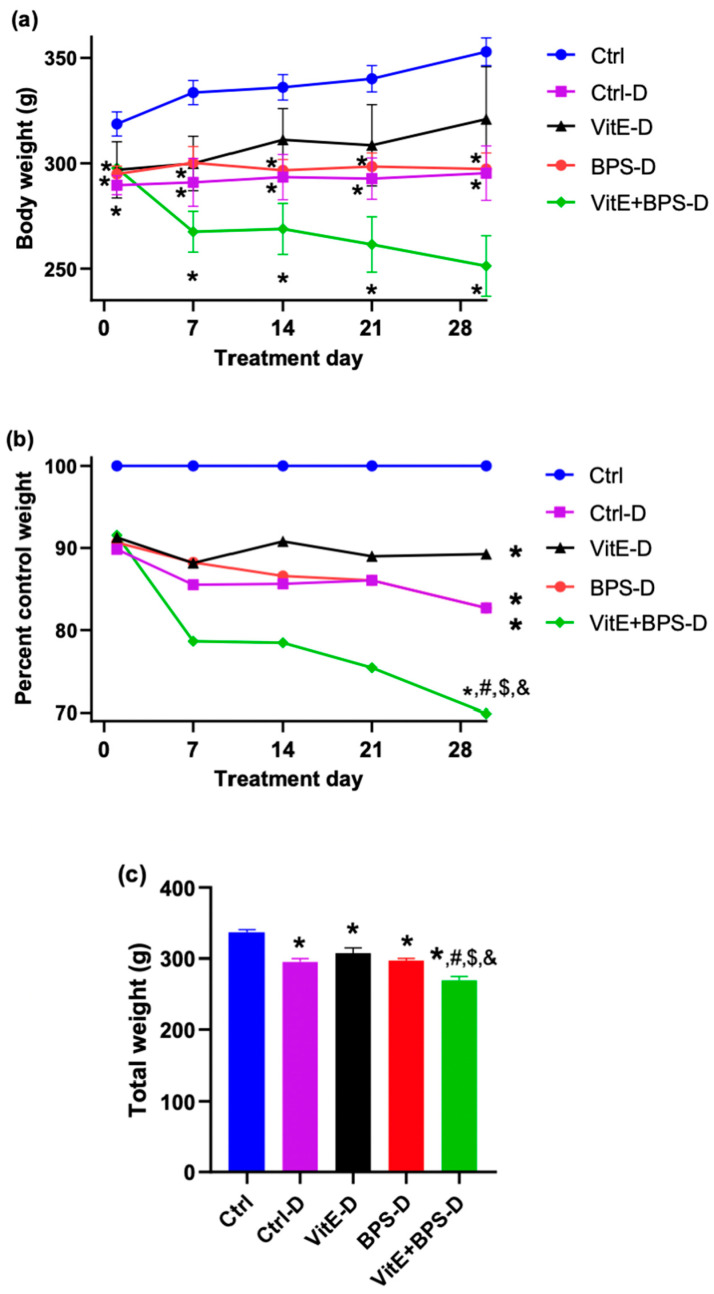
Effects of vitamin E (VitE), bisphenol S (BPS), or VitE + BPS on body weight (BW). BW was measured weekly and on day 30 of oral treatment; (**a**) Mean body weight. (**b**) The percentage of control weight. (**c**) The total weight (mean of all weights evaluated at 7, 14, 21, and 28 days). Data are expressed as mean ± SEM (*n* = 5/6 animals per group) of male-treated Wistar rats. * *p* ≤ 0.05 versus Ctrl group; ^#^ *p* ≤ 0.05 versus Ctrl-D group; ^$^ *p* ≤ 0.05 versus BPS-D group, ^&^ *p* ≤ 0.05 versus VitE-D (specific *p* values are included in the text).

The weight of the Ctrl-D group was significantly lower than that of the Ctrl group on day 0 (* *p* = 0.0035), day 7 (* *p* = 0.0155), day 14 (* *p* = 0.0123), day 21 (* *p* = 0.0049), and day 30 (* *p* = 0.0072). The weight of the BPS-D group was significantly lower than that of the Ctrl group on day 0 (* *p* = 0.0086), day 7 (* *p* = 0.0091), day 14 (* *p* = 0.008), day 21 (* *p* = 0.0013) and day 30 (* *p* = 0.0004). The weight of the VitE + BPS-D group was significantly lower than that of the Ctrl group during the treatment period (* *p* = 0.0146 on day 0; * *p* = 0.0008 on day 7; * *p* = 0.0026 on day 14; * *p* = 0.0019 on day 21, and * *p* = 0.0008 on day 30) (Figure 2a). There was no statistical difference between the VitE-D and Ctrl or the Ctrl-D groups (Figure 2a). The VitE + BPS group showed a percentage of weight loss significantly higher than that of all groups (* *p* < 0.0001, ^#^ *p* = 0.0002, ^$^ *p* = 0.0004, ^&^ *p* = 0.005 for Ctrl, Ctrl-D, BPS-D, and VitE groups, respectively). On day 30, the animals from the VitE + BPS-D group had lost almost 23% of their initial weight. In contrast, the VitE-D group maintained its initial weight. The Ctrl-D and BPS-D groups lost 8% of their body weight at 30 days of treatment (Figure 2b). The total weights of the Ctrl-D, VitE-D, BPS-D, and VitE + BPS-D groups were significantly lower than that of the Ctrl group (* *p* < 0.0001 in these comparisons). In addition, the total weight of the VitE + BPS-D group was statistically lower than those of the Ctrl-D (^#^ *p* = 0.0003), BPS-D (^$^ *p* = 0.0001), and VitE-D (^&^ *p* < 0.0001) groups (Figure 2c). Before the treatment period (at the streptozotocin administration day), the animals’ weights were similar between all groups: Ctrl (325.9 ± 5.511), Ctrl-D (315.6 ± 10.03), VitE-D (321.8 ± 8.941), BPS-D (322 ± 2.345), and VitE + BPS-D (323.6 ± 7.125). Data were evaluated by one-way ANOVA F (DFn:4, DFd:21) 0.2872, *p* = 0.8830. 

Data were evaluated by two-way ANOVA (Figure 2a): interaction: F (DFn:20, DFd:105) = 4.206, *p* < 0.0001; treatment day: F (DFn:1.619, DFd:34) = 8.068, *p* = 0.0025; group: F (DFn:4, DFd:21) = 6.780, *p* = 0.0011. Data were analyzed by generalized linear model (GLM) and Tukey Kramer post hoc test (Figure 2b). Data were evaluated by one-way ANOVA: (Figure 2c): F (DFn:4, DFn:151) = 21.05, *p* < 0.0001 (Figure 2c).

### 3.2. Enzymatic Antioxidant Activities

The activity of GPx, GST, and GR was measured in serum to determine which antioxidant enzymes are involved in the potential protective effect of VitE against damage provoked by BPS.

**Figure 3:**
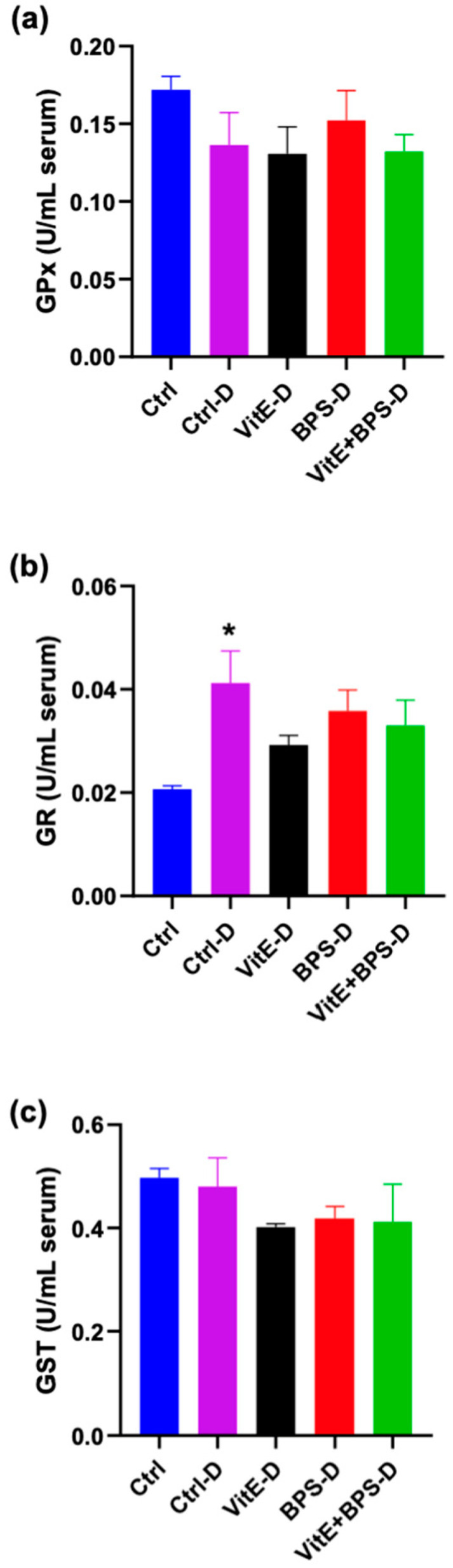
Effects of Vitamin E (VitE), bisphenol S (BPS), or VitE + BPS on (**a**) glutathione peroxidase (GPx), (**b**) glutathione reductase (GR), and (**c**) glutathione-S-transferase (GST) activity in serum of treated male Wistar rats. Data are presented as mean ± SEM, *n* = 5–6 animals per group. * *p* ≤ 0.05 versus Ctrl group (specific *p* values are included in the text).

We found that GR activity was significantly increased in animals from the Ctrl-D group compared with the Ctrl group (* *p* = 0.0011). However, BPS-D, VitE-D, and VitE + BPS-D were considered as no discovery when compared with the Ctrl group (q = 0.0521; q = 0.1097 and q = 0.1062, respectively), although the individual *p* values were 0.011; 0.0467 and 0.0337, respectively. We did not observe a significant difference between the groups when evaluating GPx and GST (Figure 3). 

Data were evaluated by one-way ANOVA (Figure 3a) F (DFn:4, DFd:21 = 1.329, *p* = 0.2921); (Figure 3b) F (DFn:4, DFd:21) = 4.059, *p* = 0.0136; (Figure 3c) F (DFn:4, DFd:20) = 1.090, *p* = 0.3881.

### 3.3. Glucose Tolerance

**Figure 4:**
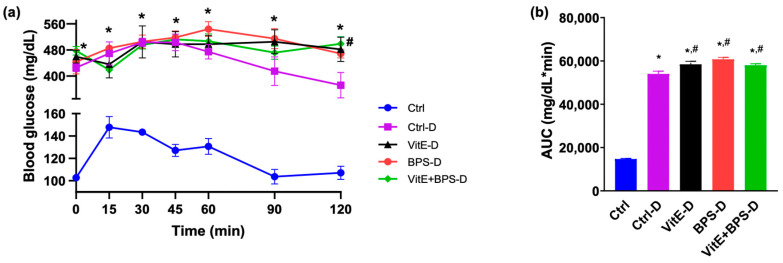
(**a**) Blood glucose concentrations (mg/dL) following administration of glucose load (2 g/kg bw) in male Wistar rats. (**b**) Mean total glucose area under the curve (AUC). Data are presented as mean ± SEM, *n* = 5–6 animals per group. * *p* ≤ 0.05, compared to the Ctrl group; ^#^ *p* ≤ 0.05, compared to the Ctrl-D group (specific *p* values are included in the text).

Blood glucose in all diabetic rat groups was higher than in the Ctrl group in 0 (* *p* ≤ 0.0001 for Ctrl-D, BPS-D, and VitE + BPS-D, and * *p* = 0.0008 for VitE-D), 15 (* *p* = 0.0005 for Ctrl-D, * *p* ≤ 0.0001 for BPS-D and VitE + BPS-D, and * *p* = 0.0018 for VitE-D), 30 (* *p* ≤ 0.0001 for Ctrl-D, BPS-D, VitE + BPS-D and * *p* = 0.0018 for VitE-D), 45 (* *p* ≤ 0.0001 for Ctrl-D, BPS-D, VitE + BPS-D and * *p* = 0.0006 for VitE-D), 60 (* *p* ≤ 0.0001 for all groups), 90 (* *p* ≤ 0.0019 for Ctrl-D, * *p* ≤ 0.0003, for VitE-D, * *p*≤ 0.0001 for BPS-D and VitE + BPS-D), and 120 min (* *p* = 0.0022 for Ctrl-D, * *p* = 0.0004 for VitE-D, * *p* ≤ 0.0001 for BPS-D and VitE + BPS-D groups). Blood glucose was higher in the VitE + BPS-D group than in the Ctrl-D group (^#^ *p* = 0.0286) (Figure 4a). The Ctrl-D, BPS-D, VitE-D, and VitE + BPS-D groups exhibited a statistically higher total glucose response (AUC values) to the glucose load relative to the Ctrl (* *p* < 0.001 in all cases). The VitE-D, BPS-D, and VitE + BPS-D groups presented statistically higher AUC values than the Ctrl-D group (^#^ *p* = 0.0035, ^#^ *p <* 0.0001, and ^#^ *p* = 0.0076, respectively). There was no difference in blood glucose levels between the VitE-D and VitE + BPS-D groups (Figure 4b). Data were evaluated by two-way ANOVA (Figure 4a): interaction: F (DFn:24, DFd:126) = 2.262, *p* = 0.0019; time (min): F (DFn:3.204, DFd:67.28) = 7.610, *p* = 0.0001; group: F (DFn:4, DFd:21) = 94.63, *p* < 0.0001; one-way ANOVA: (Figure 4b): F (DFn:4, DFn:21) = 477.4, *p* < 0.0001.

### 3.4. Biochemical Assays

Creatinine, AST, HDL-c, LDL-c, and A/G ratio are presented in Table 2. Compared with the Ctrl group, exposure to VitE + BPS significantly increased HDL-c, LDL-c, and A/G (* *p* = 0.0099, 0.0008, and 0.0028, respectively); the Ctrl-D group revealed an increase in HDL-c (* *p* = 0.0014), and A/G (* *p* = 0.005); and exposure to VitE produced an increase in HDL-c (* *p* = 0.0095). In addition, exposure to BPS produced no significant difference compared to the Ctrl in all analytes (Table 2). However, the LDL-c increased significantly in the VitE + BPS-D group versus the VitE-D group (^&^ *p* = 0.01). Regarding the reference values (RV), no alteration in creatinine or A/G levels was observed in the five groups. All groups showed higher AST and HDL-c levels than the RV, except the Ctrl group (Table 2).

**Figure 5:**
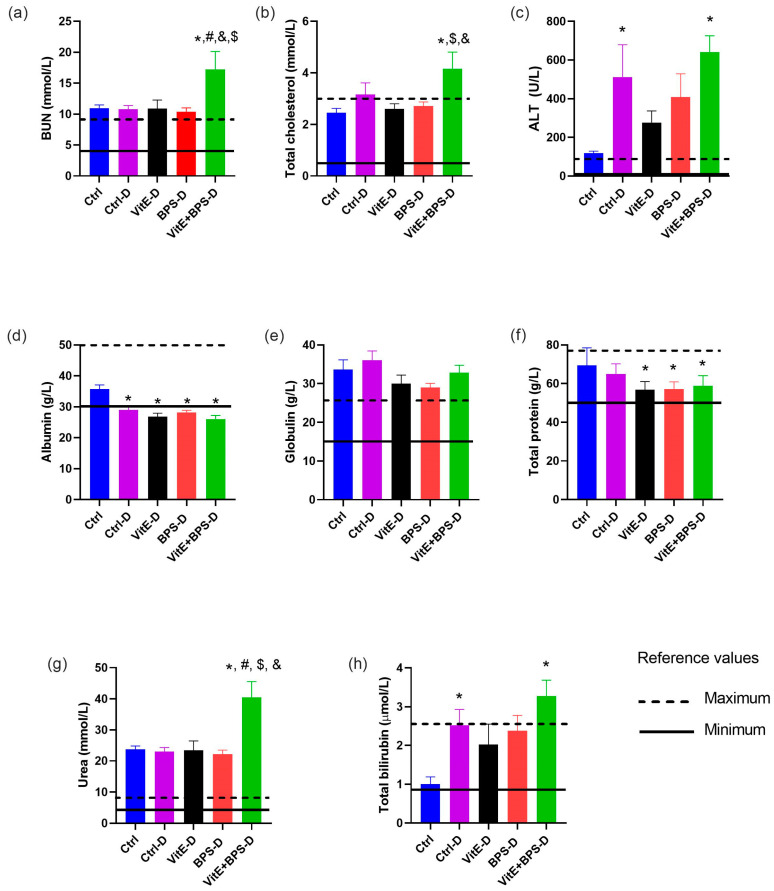
Effects of Vitamin E (VitE), bisphenol S (BPS), VitE + BPS on (**a**) blood urea nitrogen (BUN; reference value (RV): 4.02–9.13 mmol/L); (**b**) total cholesterol (RV: 0.51–2.85 mmol/L); (**c**) alanine aminotransferase (ALT; RV: 19–48 U/L); (**d**) albumin (RV: 30–50 g/L); (**e**) globulin (RV: 15–25 g/L); (**f**) total protein (RV: 56–76 g/L); (**g**) urea (RV: 4.28–8.57 mmol/L); (**h**) total bilirubin (RV: 0.86–2.57 μmol/L). Values are means ± SEM (*n* = 5/6) of male-treated Wistar rats. Solid line range: minimum reference value; dotted line: maximum reference value. * *p* ≤ 0.05 versus Ctrl group; ^#^ *p* ≤ 0.05 versus Ctrl-D; ^$^ *p* ≤ 0.05 versus BPS-D; ^&^ *p* ≤ 0.05 versus VitE-D (specific *p* values are included in the text).

BUN levels were significantly higher in the VitE + BPS-D group than in the Ctrl, Ctrl-D, VitE-D, and BPS-D groups (* *p* = 0.0048, ^#^ *p* = 0.0054, ^&^ *p* = 0.0062 and ^$^ *p* = 0.0036, respectively). Total cholesterol was significantly higher in the VitE + BPS-D than in the Ctrl, BPS-D, and VitE-D (* *p* = 0.0027, ^#^ *p* = 0.0118, ^&^ *p* = 0.0075, respectively). ALT was significantly higher in the VitE + BPS-D (* *p* = 0.001) and Ctrl-D (* *p* = 0.009) groups than in the Ctrl group (Figure 5).

The BPS, VitE, and VitE + BPS exposure statistically decreased albumin levels in diabetic animals compared with the Ctrl group (* *p* = 0.0002, * *p* < 0.0001, * *p* < 0.0001, respectively). Moreover, the Ctrl-D group also presented lower albumin levels than the Ctrl group (* *p* = 0.0005). Data established that globulin was not altered in any group. Total protein data indicated that exposure to BPS, VitE, and VitE + BPS in diabetic animals significantly decreased the levels compared to the Ctrl group (* *p* = 0.0033, 0.0026, 0.009, respectively). Urea levels were significantly higher in animals from group VitE + BPS-D than in the Ctrl, Ctrl-D, VitE-D, and BPS-D groups (* *p* = 0.0002, 0.0002, 0.0003 and 0.0001, respectively). Total bilirubin levels were significantly higher in animals from the VitE + BPS-D and Ctrl-D groups compared to animals from the Ctrl group (* *p* = 0.0004, 0.0104, respectively) (Figure 5).

The Ctrl group was in the RV in total cholesterol, protein, bilirubin, and albumin. The values for BUN and ALT analytes of the Ctrl group were slightly higher than the RV. However, urea and globulin levels in this group were at least twice the value of the minimum Ctrl RV. All treated groups presented, as compared to the RV, higher levels in the ALT and globulin analytes, in contrast with the albumin, where all experimental groups were lower than the RV. The VitE + BPS-D group was the one that presented more alterations as compared to the RV in BUN, total cholesterol, ALT, albumin, urea, and total bilirubin (Figure 5).

Data were evaluated by one-way ANOVA: Creatinine, F (DFn:4, DFd:21) = 0.3682, *p* = 0.8285; AST, F (DFn:4, DFd:20 = 1.592, *p* = 0.2134 ); HDL-c, F (DFn:4, DFd:21) = 4.053; *p* = 0.0137; LDL-c, F (DFn:4, DFd:21) = 4.064, *p* = 0.0136; Albumin /globulin, F (DFn:4, DFd:21) = 3.902, *p* = 0.0160; BUN, F (DFn:4, DFd:21 = 3.914, *p* = 0.0158); Total cholesterol, F (DFn:4, DFd:21) = 3.562, *p* = 0.0228; ALT, F (DFn:4, DFd:21); = 4.382, *p* = 0.0099; Albumin, F (DFn:4, DFd:21) = 11.51, *p*< 0.001; Globulin, F (DFn:4, DFd:21) = 1.684, *p* = 0.1911; Total protein, F (DFn:4, DFd:21) = 4.564, *p* = 0.0083; Urea, F (DFn:4, DFd:21) = 7.948, *p* = 0.0005; Total bilirubin, F (DFn:4, DFd:21) = 4.788, *p* = 0.0067.

### 3.5. Pancreatic Histology

**Figure 6:**
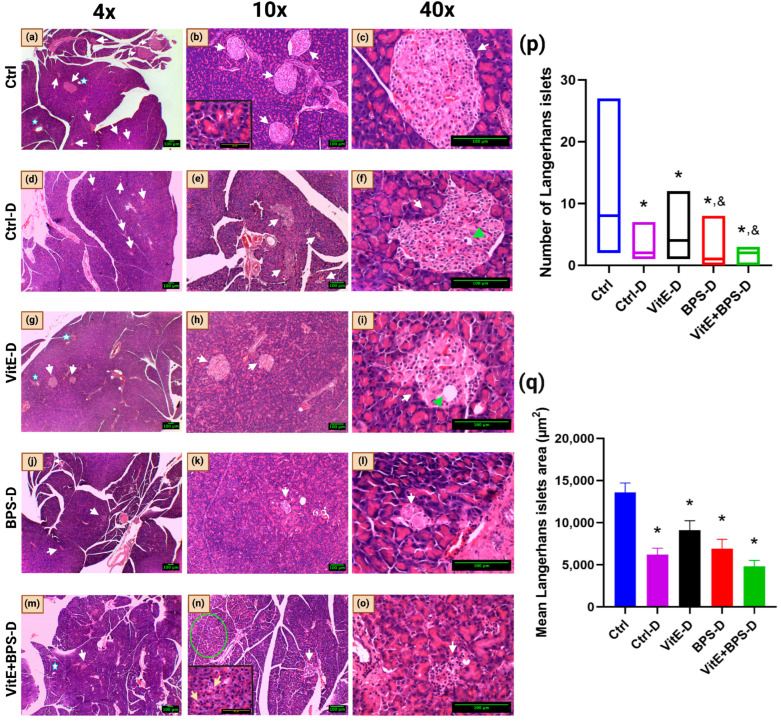
Effects of vitamin E (VitE), bisphenol S (BPS), or VitE + BPS on pancreas histology, stain with H&E. Bar = 100 µm. Photomicrographs of pancreatic tissue in 4×, 10×, and 40× magnification of (**a**–**c**) Ctrl group; (**d**–**f**) Ctrl-D group; (**g**–**i**) VitE-D group; (**j**–**l**) BPS-D group; (**m**–**o**) VitE + BPS-D group. Inset in (**b**) indicates the 100× magnification of the exocrine parenchyma of the Ctrl group. Inset in (**n**) denotes the 100× magnification of the exocrine parenchyma of the VitE + BPS-D group; yellow arrows indicate vacuolization inside of cells. (**p**) Boxplot of the number of Langerhans islets; * *p* ≤ 0.05 versus Ctrl and ^&^ *p* ≤ 0.05 versus VitE-D. (**q**) Mean Langerhans islets area (µm^2^); * *p* ≤ 0.05 versus Ctrl (specific *p* values are included in the text). White arrows indicate Langerhans islets; blue stars show the pancreatic duct; the green circle exhibits the vacuolization and degeneration of the exocrine pancreas; the green arrowhead displays the lipid infiltration in Langerhans islets cells.

The histological examination of the Ctrl group for endocrine and exocrine hematoxylin and eosin-stained pancreas indicated no apparent pathological changes and a typical appearance of the pancreatic tissue. The exocrine pancreas of the Ctrl-D group displayed vacuolization, and the endocrine pancreas presented a reduction in Langerhans islets area and number as compared to the Ctrl (* *p* = 0.001, *p* < 0.00001, respectively), tissue degeneration, and lipid infiltration. In the VitE-D group, the exocrine pancreas did not display any apparent pathological changes, like in the Ctrl group. Regarding the endocrine pancreas, a decrease in the area and the number of Langerhans islets (* *p* = 0.018, 0.0002, respectively), lipid infiltration, and slight tissue degeneration were observed compared to the Ctrl group. The exocrine pancreas of animals from the BPS-D group exhibited slight to moderate degeneration; the endocrine pancreas presented degeneration and decreased Langerhans islets area and number compared to the Ctrl group (* *p* = 0.007, * *p* < 0.00001, respectively), and decreased number of Langerhans islets compared to the VitE-D group (^&^ *p* = 0.014) and lymph node histiocytosis. The exocrine pancreas of the VitE + BPS-D group exhibited slight to moderate vacuolization and lymph node edema. Although the Langerhans islets in two animals of this group presented atrophy, the other three presented no apparent pathological changes in the endocrine pancreas. In addition, the Langerhans islets number was lower than in the Ctrl and VitE-D groups (* *p* < 0.00001, ^&^ *p* = 0.005, respectively), and its mean area was significantly lower than in the Ctrl (* *p* = 0.0004) (Figure 6, Table 3). Data were analyzed by GLM and Tukey Kramer post hoc test (Figure 6p,q). 

### 3.6. Urinalysis

We performed a urinalysis to evaluate the alterations in renal metabolism produced by administering VitE, BPS, and their combination. The highest turbidity in the urine samples was observed in the samples from the BPS-D group, where two animals exhibited a value of 2+, and one 1+. In the VitE + BPS-D group, one animal presented a value of 1+, and another showed 2+. One animal from the VitE-D group had a 2+ value. Two animals from the Ctrl-D group exhibited 1+. One animal from the Ctrl group displayed a 1+ value.

All animals had negative nitrites, urobilinogen, red blood, and ketones. Two animals from the Ctrl-D group and one from the VitE + BPS-D group showed 17 pmol/L of bilirubin. In the other animals, the result was negative. The specific gravity in the Ctrl-D, VitE-D, BPS-D, and VitE + BPS-D groups was significantly higher than in the Ctrl group (* *p* < 0.0001, * *p* = 0.0004, * *p* < 0.0001, * *p* < 0.0001, respectively). That in the VitE-D group was significantly lower than in the BPS-D (^$^ *p* < 0.0004) and Ctrl-D groups (^#^ *p* < 0.0264). The VitE + BPS-D group presented specific gravity values similar to that in the Ctrl-D group but significantly different than those in the Ctrl, BPS-D, and VitE groups (* *p* < 0.0001, ^$^ *p* = 0.0404, ^&^ *p* = 0.0498, respectively) (Table 4).

Regarding glucose, we observed that the values in the Ctrl-D, VitE-D and VitE + BPS-D groups were significantly higher than that in the Ctrl group (* *p* = 0.008, 0.26, 0.0003, respectively). The glucose in the VitE + BPS-D group was higher than in the BPS-D group (^$^ *p* = 0.21) (Table 3). The highest leukocyte count was observed in the VitE-D group compared with the Ctrl group (* *p* = 0.035), and the lowest count was observed in the VitE + BPS-D group. All groups had no significant difference in urine pH value or protein content (g/L). The one-way ANOVA F (DFn:4, DFd:20) = 21.23, *p* < 0.0001 evaluated the specific gravity; pH F (DFn:4, DFd:20) = 2.746, *p* = 0.0571. Leukocytes (cells/μL), protein (g/L), and glucose (mmol/L) were evaluated by GLM and Tukey post hoc tests.

Data are expressed as mean ± SEM. * *p* ≤ 0.05, vs. Ctrl; ^#^ *p* ≤ 0.05 vs. Ctrl-D; ^&^ *p* ≤ 0.05 vs. VitE-D; ^$^ *p* ≤ 0.05 vs. BPS-D (*n* = 5 for group).

### 3.7. Nutrient Absorption and Digestibility

We conducted a PCA to evaluate the effects of VitE, BPS, and their combination on nutrient absorption and digestibility since both compounds are absorbed partially in the intestine [50,51]. The values obtained in the PCA of the food administered to the animals were like the ones reported by the food producers (FS) (BIO-DIETA-LAB 7300 (ABENE^®^, Mexico), in their guaranteed analysis. The Ctrl group exhibited the highest percentage of raw protein, fat, ash, AIA, and digestibility in the stool. At the same time, the nitrogen-free extract (NFE) was lower than in the other groups. Diabetic rats (Ctrl-D, VitE-D, BPS-D, and VitE + BPS-D groups) absorbed more nutrients than the Ctrl group, as indicated by decreased raw protein, raw fat, and ash. Still, the digestibility was lower. Animals from the VitE-D and VitE + BPS-D groups showed values closest to the Ctrl group in raw protein, fat, and ash (Table 5). Pool data was presented as percent.

## 4. Discussion

Exposure to environmental chemicals such as bisphenols causes potential risks, including obesity and metabolic disorders such as DM, which is a chronic disorder that leads to alterations in the intracellular metabolism and hyperglycemia that alters insulin responsiveness and hepatic gluconeogenesis, provoking hormonal disturbances [52,53].

The present research aimed to evaluate whether exposure to BPS induces alterations in the structure and function of the pancreas, activity of serum antioxidant enzymes, absorption of nutrients, and liver and kidney functions and if coadministration with VitE could protect from the harmful effects produced by BPS in a Wistar male rat diabetic model. In this study, we demonstrated that BPS in diabetic rats (BPS-D group) decreased the tolerance to glucose, plasma albumin, and total proteins compared to the control (Ctrl group). Additionally, the VitE coadministration with BPS aggravated the damage in the liver and kidney of male rats according to an increase in BUN, total cholesterol, ALT, urea, and total bilirubin.

Body weight is one of the indicators of animals’ general health condition [54]. Furthermore, it is known that one of the effects of DM is a decrease in body weight [55]. Accordingly, all diabetic animals presented a lower body weight than the Ctrl animals.

At the end of the experiment, the animals from the VitE-D group had gained about 24 g from their initial weight, and the Ctrl-D group had gained around 3 g. This weight gain is consistent with some publications that suggest that VitE reduces the characteristic symptoms of diabetes, such as weight loss [56]. Contrarily, Shamsi et al. (2004) reported that oral administration of VitE for 3 weeks induced weight loss in healthy and diabetic rats [30]. Also, there are reports where VitE administration does not produce alterations in body weight [57,58]. Thus, the difference between the results is probably due to the different doses of VitE used since there are reports that the effects of VitE are dose-dependent [30]. Regarding BPS, and in concordance with our results, in a study performed with Wistar rats treated with BPS, there were no differences in rat body weight [59]. Contrarily, male Sprague–Dawley rats with diabetes induced with streptozotocin and administered BPA showed a decrease in body weight; in the same study, administering the same dose of BPA in healthy rats increased this parameter [60]. Thus, the effect of bisphenols depends on the amount, health condition, and age at the time of administration.

Concerning the body weight, the VitE + BPS-D group lost around 46 g compared with their initial weight and presented the lowest weight compared to all other groups. Thus, rats in the VitE + BPS-D group were more susceptible to weight loss when BPS and VitE were concomitantly administered. On the other hand, there were no significant differences in total body weight between the Ctrl-D, BPS-D, and VitE-D groups. It is possible that administering BPS and VitE separately may not reduce weight, but their combined use may have a more significant impact.

BPS has been demonstrated to act as an endocrine-disrupting compound [61]. Nevertheless, in the literature, most BPS toxicity studies have focused on the reproductive effects [62,63]. However, besides its inherent effects on the reproductive system, BPS is also known to alter glucose levels [64] and oxidative stress by affecting antioxidant activity.

The OGTT currently serves as the gold standard for evaluating glucose metabolism [65,66]. In the present study, OGTT results revealed increased blood glucose concentrations in the BPS-D, VitE-D, and VitE + BPS-D groups, as compared to the Ctrl and Ctrl-D groups, indicating abnormal metabolism of glucose, which remains in the bloodstream. Additionally, the effect of BPS on increasing blood glucose concentration has been reported in other studies. Mandrah et al. (2020) [59] reported that in Wistar rats, a hyperglycemic condition was initiated by sub-chronic exposure to BPS [59]. In contrast, a significant increase in glucose tolerance was reported in 36-day-old Wistar male rats perinatally treated with BPS [64]. The differences between the two studies may be due to the period in which the animals were exposed to BPS and the administration method.

In the present study, VitE did not decrease the glucose levels in diabetic rats, as evaluated through AUC values. It has been reported that the effects of VitE on blood glucose depend on the dose and the metabolism state [67]. VitE decreased blood glucose levels in diabetic models, such as T1DM Wistar rats [30] and humans [68]. In contrast, other studies reported that VitE did not improve the regulation of glucose-stimulated insulin secretion in hyperglycemic mice [69] and did not change blood glucose levels in diabetic patients [70].

Administration of a single dose of STZ selectively destroys β-cells in the pancreas and produces a permanent diabetic state [71]. In the present study, the alterations observed in the pancreas histology in the diabetic experimental groups were expected: vacuolization, reduction of Langerhans islet area and number, tissue degeneration, and lipid infiltration [72]. However, an increase in pancreatic damage was observed in the BPS-D group compared to the Ctrl and Ctrl-D groups. Vacuolization is one of the first alterations recognized when there has been cell damage and corresponds to the accumulation of endogenous substances, a product of the alteration in cell metabolism [48,49]. If the injury is prolonged, it will produce cell degeneration and can later end in cell death, which is observed in reducing the number and area of Langerhans islets. BPS escalated the damage in the pancreas, provoking degeneration of both the endocrine and exocrine pancreas [73,74]. However, the coadministration of VitE with BPS protected, to some extent, the endocrine pancreas since no apparent pathological changes were observed in three of five animals from this group. In agreement with our results, it has been previously reported that VitE improves the histoarchitecture of the pancreas in diabetic animals [75,76].

Bisphenols’ effects depend on several factors, such as the animal model, the dose, the period of administration, etc. Bisphenols affect pancreatic β-cell insulin content and secretion in a non-monotonic dose–response manner (NMDR) [77]. In contrast to our results, the administration of BPA to rats for eight weeks did not induce histological alterations or differences in the size of Langerhans islets [78]. Supplemented VitE participates in the inflammatory response mechanisms in the pancreas and has anti-inflammatory and beneficial effects [79].

The primary route of human exposure to BPS is through food and liquids stored in plastic containers [54]. After oral ingestion, BPS passes to the intestine and the liver, where it is metabolized in animals and humans [80,81]. The liver is a critical tissue for maintaining glucose and cholesterol homeostasis [82] and transforming bisphenols into a glucuronic-conjugated form [54]. Thus, the liver is an organ vulnerable to BPS toxicity.

The results from our study suggest that the liver was one of the organs extremely affected by the coadministration of VitE and BPS. The VitE + BPS-D group showed significant changes from the RV in 12 of the 14 analytes examined. However, compensatory actions such as HDL-c increase were observed, with no improvement due to the parallel rise in LDL-c, which is associated with liver failure (bilirubin, AST, and ALT) [83]. In addition, animals of the VitE + BPS-D group showed a significant increase compared with those in the other experimental groups in BUN, total cholesterol, ALT, and urea. The increase in hepatic enzymes observed in the present article was like that in other studies evaluating BPS’ effect in Wistar rats [81], where the authors considered that BPS produced potential hepatic intoxication [81]. In addition, the effects caused by BPS on the liver function observed in the present research were similar to those caused by BPA [84,85].

The Ctrl-D, VitE-D, BPS-D, and VitE + BPS-D groups showed a decrease in albumin and total protein compared to the Ctrl group. Serum proteins were expected to decrease in the diabetic animals compared with the Ctrl group because, in a diabetic condition, the serum proteins are lost in the urine [86,87]. However, the total protein in the VitE-D and BPS-D groups also showed a significant decrease (*p* ≤ 0.05) compared with the Ctrl-D group. Thus, it is necessary to evaluate the function of VitE and BPS in the damage produced by the diabetic condition. The total cholesterol was increased significantly in animals from the VitE + BPS-D group versus those in the Ctrl, BPS-D, and VitE-D groups. Thus, VitE + BPS-D probably alters sex hormone levels in the blood, exacerbating the hepatic steroid alterations that occur in a diabetic state [71,88].

After the Ctrl group, the Ctrl-D group revealed levels closest to the reference values. It has been reported that when T1DM is induced pharmacologically in an experimental rat model, the animals present changes in serum chemistry and liver enzymes [30,40] due to disorders in the metabolism of carbohydrates, fats, and proteins [89]. These alterations are consistent with the changes observed in the Ctrl-D group’s HDL-c, ALT, and total bilirubin. However, we observed significant alterations between experimental groups treated with STZ; we consider that the effects of BPS and the coadministration of VitE and BPS are independent of the effect of STZ and could be related to kidney failure and liver overwork.

Regarding the VitE + BPS-D group, LDL-c was significantly higher than in the Ctrl and VitE-D groups and about 0.3 mmol/L above the Ctrl-D and BPS-D values. VitE is an antioxidant that protects the hepatic cells against oxidative stress and prevents fatty liver disease [90]. It has been described that supplementation with VitE decreased LDL-c levels, total plasma cholesterol, triglycerides, and oxidative susceptibility in patients with DM [91]. Additionally, in another study, VitE decreased the same parameters in diabetic rats [92]. Therefore, the difference between our results and the mentioned studies could be because of a difference in the extent of the experiment.

In the PCA, there was a decrease in the percentage of nutrients quantified between the groups of rats with DM compared with the Ctrl group (raw protein, raw fat, and ash). This could be due to the metabolic demands of the sick subjects under experimentation; rats without metabolic alterations do not require nutrients available in the diet beyond those used for homeostatic balance. On the other hand, rats with DM require essential nutrients to compensate for the metabolic state of disease triggered by diabetes, using physiological digestive mechanisms and adaptations to absorb elements in the diet [93,94,95]. For example, studies suggest that in rats, diabetes induced by streptozotocin is connected to a rise in glucose absorption through the small intestinal mucosa [94,95]

The observed out-of-range values in the Ctrl group in the serum biochemistry could be associated with a diet rich in fat, possibly produced by the oil used as the vehicle and the high protein in the diet. According to dietary recommendations for rodents, a diet with around 14% protein is considered ideal for their organic maintenance [96]. In our study, the percentage of protein was 35% because diabetic animals needed more nutrients.

It has been reported that i.p. administration of STZ in male Wistar rats induced uncontrolled diabetes and increased endogenous biliary excretion and plasma bilirubin, as well as enhanced hepatic conjugation and subsequent biliary excretion of the pigment [71]. Similarly, we observed that the serum levels of bilirubin in all experimental groups were increased compared to the Ctrl group. Still, this increase was only significantly different from the Ctrl in the VitE + BPS-D and Ctrl-D groups and was out of the reference range. Thus, the effect of the coadministration of VitE + BPS on the hepatic transport and excretion of bilirubin in streptozotocin-induced diabetes male Wistar rats requires further investigation.

Since the harmful effects of bisphenols are of great importance for human and animal health, and the complications of DM may be linked to oxidative stress [67], efforts have been made to propose alternatives to counteract or decrease the negative consequences of their use [97,98,99,100].

In healthy rats, the effects of coadministration of vitamins with bisphenols are contradictory; i.e., male rats treated with VitE showed improved kidney function tests and alleviated BPA-induced oxidative stress in the kidney and nephrotoxicity [33]. In adult male Wistar rats, the administration of VitE had a protective effect on the administration of BPA (in reproductive parameters) [25]. In contrast, administering vitamin C aggravated bisphenol damage in histopathological lesions in the testis [27]. Korkmaz et al. [101,102] demonstrated that the coadministration of vitamin C with BPA augments kidney and liver damage in male rats [101,102]. Vitamin C loses its effectiveness as an antioxidant at high concentrations. However, it can act as a pro-oxidant in tissues, reducing metals to forms that react with oxygen that produce initiators of lipid peroxidation [103]. Also, excess VitE can produce dangerous effects by disrupting the detoxification chain of oxidative stress, and radicals will remain with pro-oxidant properties [104,105].

Mammalian cells have enzymatic and non-enzymatic antioxidant defense mechanisms that reduce cell injury produced by interacting lipids, proteins, DNA molecules, and reactive oxygen species (ROS). Under normal metabolism, the continuous formation of ROS and other free radicals is essential for normal physiological functions such as ATP production, catabolic and anabolic processes, and cellular redox cycles [106]. In DM, oxidative stress is increased due to the impairment in the antioxidant defense and increased oxidation of proteins or lipids. Hyperglycemia is an inducer of ROS and nitrogen species [107]; it increases the formation of advanced glycosylation products that result from the reaction of glucose and other monosaccharides with proteins, producing chemically and biologically modified molecules that lead to various imbalances [56]. Moreover, it activates the protein kinase C, producing changes in cell permeability [108]. The development of the chronic complications of DM may be linked to oxidative stress [109,110,111]. Thus, an unbalanced production of ROS in cells plays a role in the pathogenesis of DM [14].

We evaluated the antioxidant enzymes because it has been reported that BPA-induced toxicity is closely linked to the impairment of oxidant–antioxidant systems balance, altering GSH content as well as superoxide dismutase and catalase levels in the blood and the function of many vital organs and cells, such as the liver, kidney, testes, and pancreas, accompanied by increased production of ROS, mitochondrial dysfunction, and modulation of cell signaling pathways [14,112,113]. Antioxidants are present in all body fluids and may interact with the free radicals produced by bisphenol exposure and disrupt the sequence of oxidation reactions before DNA, RNA, lipids, and proteins are damaged [14,114]. This research did not detect differences between GPx, GR, and GST activity in the serum in the VitE-D, BPS-D, and VitE + BPS-D groups versus the Ctrl and Ctrl-D groups. Thus, we consider that the alterations in GR observed in the diabetic experimental groups versus the Ctrl group (healthy rats) are expected because of the alteration in the antioxidant defense in diabetes as mentioned above.

In diabetic animals, there are alterations in drug metabolism because there are reduced concentrations of cytochrome P450 and impairment of some phase II pathways, such as glucuronidation or conjugation [115,116]. VitE and BPS metabolism are very similar in humans and animals [117]. The metabolism of BPS is generally performed by liver cytochrome P450 and CYP3A4 enzymes [118,119,120,121]; in addition, VitE is metabolized as a xenobiotic by the cytochrome P450 and increases the expression of cytochrome P-450-3A [104,122]; its metabolism uses liver-binding proteins, which selectively bind α-tocopherol. The α-tocopherol transfer protein mediates its transport from the liver to lipoproteins to maintain homeostatic control of α-tocopherol concentrations in blood and tissues [117,123]. Therefore, the effects reported in this article may be due to an additive effect of VitE on the toxicity of BPS because some reports indicate that VitE and BPS or their metabolites accumulate in the liver [122,124,125].

Some studies have reported the beneficial effects regarding the coadministration of VitE with bisphenols in other organs, such as the testes [126], the lungs [127], and the kidneys [33]. The difference in these studies is that they used healthy rats with the regular activity of cytochrome P450, and the animals may have been capable of metabolizing both compounds. However, it was reported that VitE could produce beneficial effects and be safe in some organs, such as the testes, because it is essential for normal spermatogenesis but, at the same time, not safe for the liver and kidneys. This must, therefore, be considered when using or recommending VitE for treatment purposes [128].

Considering the concentrations of antioxidants and bisphenols used in different animal models is essential. Because bisphenols do not produce a monotonic response [61], the VitE effects depend, among other factors, on the quantity of lipids in the body. We agree with Mączka et al. (2022), who mention that it is necessary to account for the influence of bisphenols on the metabolism and the power of doses of antioxidants used to attenuate their harmful effects [14]. Although there is limited information about the impact of antioxidants on BPS effects, our results support the results that indicate that BPA toxicity may be aggravated by metabolic disorders and coexisting diseases [14]. However, a gap remains to be investigated between the roles of the coadministration of VitE with BPS in the pathogenesis of DM.

## 5. Conclusions

Approaches using antioxidants to counteract the adverse effects of bisphenols and DM have been considered in the last decade. Most of the works directed at evaluating antioxidants’ protective effects against BPA harmful effects showed promising results. However, there are other reports where the antioxidants did not attenuate BPA toxicity effects; on the contrary, they exacerbated the damage produced by BPA. Furthermore, there are limited studies that evaluate BPS. In this article, we demonstrated that coadministration of VitE with BPS alters glucose levels, pancreas histology, and serum metabolites related to liver and kidney functionality, exacerbating the toxicity of BPS in diabetic rats. Therefore, it is necessary to consider that the replacement of BPA by its analogs, such as BPS, is inherent. Evaluating the attenuating effects of antioxidants on the toxicity produced by these BPA analogs is a field of research that is necessary to continue to determine the optimal dosage and treatment regimen of the antioxidants to counteract bisphenol toxicity.

## Figures and Tables

**Table 1:** Information on kits used for biochemical assays.

Brand	Name	Catalog Number	Lot	Expiration
SEKISUI Diagnostics^®^	Albumin	200-05	59489	29 February 2024
	ALT	318-30	61318	11 February 2023
	AST	319-10	60989	8 April 2023
	BUN	283-30	61232	31 July 2023
	Creatinine	221-30	58181	31 August 2023
	HDL-c	1001-80	59195	27 January 2023
	LDL-c	1014-80	58142	6 October 2022
	Total protein	200-55	61229	30 November 2024
SYNER-MED^®^	Total bilirubin	IR701	242105	24 May 2023

ALT: alanine aminotransferase, AST: aspartate aminotransferase, BUN: blood urea nitrogen, HDL-c: high-density lipoprotein cholesterol, LDL-c: low-density lipoprotein cholesterol.

**Table 2:** Biochemical assays in serum.

Analyte	RV	Ctrl	Ctrl-D	VitE-D	BPS-D	VitE + BPS-D
Creatinine (μmol/L)	09.00–70.00	58.50 ± 3.32	55.40 ± 3.01	52.20 ± 1.59	56.40 ± 3.44	58.60 ± 7.90
AST (U/L)	46.00–245.0	213.17 ± 17.99	379.20 ± 92.38	283.00 ± 53.85	349.40 ± 93.67	423.50 ± 82.12
HDL-c (mmol/L)	0.6–0.75	0.69 ± 0.05	1.16 ± 0.10 *	1.05 ± 0.08 *	0.95 ± 0.05	1.05 ± 0.15 *
LDL-c (mmol/L)	0.49–0.05	0.36 ± 0.03	0.50 ± 0.11	0.47 ± 0.04	0.54 ± 0.02	0.80 ± 0.15 *^,&^
Albumin /globulin	0.44–2.68	1.08 ± 0.04	0.82 ± 0.07 *	0.92 ± 0.08	0.98 ± 0.02	0.80 ± 0.06 *

Data are expressed as mean ± SEM. AST: aspartate aminotransferase; HDL-c: high-density lipoprotein cholesterol; LDL-c: low-density lipoprotein cholesterol. RV: reference value. * *p* ≤ 0.05, versus Ctrl group. VitE + BPS-D versus VitE-D: ^&^ *p* ≤ 0.05 (specific *p* values are included in the text).

**Table 3:** Pancreas histological evaluation.

Group	ID	Langerhans Islets Atrophy	Endocrine Degeneration	Endocrine Lipid Infiltration	Exocrine Degeneration	Exocrine Vacuolization	Findings
Ctrl	1	−	−	−	−	−	NF
	2	−	−	−	−	−	NF
	3	−	−	−	−	−	NF
	4	−	−	−	−	−	NF
	5	−	−	−	−	−	NF
	6	−	−	−	−	−	NF
Ctrl−D	1	+++	++	++	−	++	NF
	2	+++	++	−	−	++	NF
	3	++	++	++	−	++	NF
	4	++	−	−	−	−	NF
	5	+++	−	−	−	−	NF
VitE−D	1	+	−	−	−	−	NF
	2	−	−	++	−	−	NF
	3	−	+	+	−	−	NF
	4	++	−	−	−	+++	LE
	5	−	+	−	−	−	NF
BPS−D	1	−	−	−	+	−	NF
	2	++	−	−	+	−	NF
	3	−	++	−	+	−	LNH
	4	++	−	−	−	−	NF
	5	−	++	−	++	−	NF
VitE + BPS−D	1	++	−	−	+++	−	NF
	2	−	−	−	++	−	NF
	3	++	−	−	−	++	NF
	4	−	−	−	+	−	LE
	5	−	−	−	−	−	LE

ID: Animal identification; LE: Lymph node edema; LNH: Lymph nodes histiocytosis; NF; No Findings; −: no damage; +: scarce damage; ++: moderate damage; +++: severe damage.

**Table 4:** Urinalysis test data.

Analyte	Ctrl	Ctrl-D	VitE-D	BPS-D	VitE + BPS-D
Specific gravity	1.01 ± 0.00	1.06 ± 0.00 *	1.04 ± 0.01 *^,#,$^	1.07 ± 0.00 *	1.05 ± 0.01 *^,$,&^
Leukocytes (cells/μL)	6.00 ± 3.67	92.00 ± 13.47	178.00 ± 22.00 *	103.00 ± 83.27	59.00 ± 13.47
pH	6.90 ± 0.25	5.50 ± 0.27	5.90 ± 0.40	5.80 ± 0.34	5.60 ± 0.40
Protein (g/L)	0.14 ± 0.04	0.10 ± 0.00	0.26 ± 0.04	4.38 ± 3.90	0.10 ± 0.00
Glucose (mmol/L)	6.00 ± 3.67	48.00 ± 7.35 *	42.00 ± 7.35 *	27.00 ± 3.00	64.00 ± 12.88 *^,$^

Data are expressed as mean ± SEM. * *p* ≤ 0.05, vs. Ctrl; ^#^ *p* ≤ 0.05 vs. Ctrl-D; ^&^ *p* ≤ 0.05 vs. VitE-D; ^$^ *p* ≤ 0.05 vs. BPS-D (*n* = 5 for group).

**Table 5:** Proximate chemical analysis in pellets and stools.

Nutrient	FS	Food	Ctrl	Ctrl-D	VitE-D	BPS-D	VitE + BPS-D
Humidity (%)	12.0	11.03	56.47	60.95	46.64	57.60	46.05
Dry matter (%)	88.0	88.97	43.53	39.05	53.36	42.40	53.95
Raw protein ^1^ (%)	23.5	21.10	12.45	8.77	10.06	8.51	10.47
Raw fat ^1^ (%)	6.0	5.47	2.59	1.29	1.92	1.47	1.89
Ash ^1^ (%)	8.0	9.67	13.01	9.04	11.91	9.47	11.77
Raw fiber ^1^ (%)	4.0	5.58	3.97	3.99	4.51	3.53	5.05
NFE ^1^ (%)	46.5	47.15	11.51	15.96	24.96	19.42	24.77
AIA (%)	-	0.84	2.31	1.10	2.07	1.62	1.70
Digestibility (%)	-	-	83.84	43.84	79.62	68.35	70.79

Data are expressed as a percent of pools in each group. ^1^ Results expressed on a wet basis. AIA: Ash insoluble in acid; FS: Guaranteed analysis of food sellers; NFE: Nitrogen-free extract; PCA: Proximate chemical analysis.

## Data Availability

Data are available from the corresponding author upon reasonable request.
